# The Impact of Preconceptional Serum TSH Levels between 2.5 and 4.0 mIU/L on Infertile Women Going through Their First IUI Treatment Cycle

**DOI:** 10.1155/2019/8492904

**Published:** 2019-12-18

**Authors:** Yuchao Zhang, Wenbin Wu, Yanli Liu, Xingling Wang, Liting Jia

**Affiliations:** ^1^Department of Reproductive Medicine, The Third Affiliated Hospital of Zhengzhou University, Zhengzhou, Henan, China; ^2^Neonatal Screening Center, The Third Affiliated Hospital of Zhengzhou University, Zhengzhou, Henan, China

## Abstract

**Objective:**

To evaluate the impact of preconceptional serum TSH levels on the clinical outcomes of infertile patients undergoing their first IUI treatment cycle.

**Method:**

This was a retrospective study. Euthyroid patients undergoing the first cycle of IUI treatment from Jan 2017 to Aug 2018 were included. The patients were divided into a normal high TSH level (2.5–4.0 mIU/L) group and a low TSH level (0.4–2.5 mIU/L) group. Then, different factors were included separately to compare the outcomes between normal high and low TSH levels. The primary outcomes were clinical pregnancy rates, implantation rates, and miscarriage rates. The secondary outcomes were obstetric outcomes such as single live birth rates, birth length, birth weight, and duration of gestation.

**Results:**

Initially, 1856 patients were included, and 371 patients were excluded for different reasons. A total of 1485 patients were finally included in the analysis. The general parameters between normal high and low TSH levels were similar except for infertile duration and female BMI, which were, however, significantly different between AID-IUI patients and AIH-IUI patients (*P*=0.005 and *P*=0016). No significant differences were found in terms of either primary outcomes or secondary outcomes.

**Conclusion:**

Normal high-serum TSH levels within the reference range (0.4–4.0 mIU/L) measured before treatment seemed to have no adverse impact on the clinical outcomes of patients undergoing their first IUI treatment cycle.

## 1. Introduction

Subclinical hypothyroidism (SCH) was defined as a thyrotropin (TSH) level greater than the upper limit of normal range (which might range from 2.5 to 5.5 mIU/L according to different studies) with normal free thyroxine (FT4) levels [1–3]. Despite differences in the methods used in testing TSH and populations included as study objects among the previous studies, the American Society for Reproductive Medicine (ASRM) concluded that insufficient evidence was shown to clarify the association between TSH levels (2.5–4.0 mIU/L) and miscarriage, and that no solid data were available to evaluate whether TSH levels (2.5–4.0 mIU/L) are associated with adverse obstetric outcomes [4]. For that reason, the aim of this study was to evaluate the impact of preconceptional TSH levels between 2.5 and 4.0 mIU/L on the clinical outcomes of intrauterine insemination (IUI), which is the first-line treatment provided for infertile couples seeking help from assisted reproduction technology (ART).

## 2. Materials and Methods

### 2.1. Study Design and Population Selection

This was a retrospective study. Infertile couples (*n* = 1856) undergoing their first IUI cycle in the Department of Reproductive Medicine, the Third Affiliated Hospital of Zhengzhou University, from Jan 2017 to Aug 2018, were initially included. The IUI treatments of 39 women were cancelled due to low-quality sperm, follicular dysplasia, and personal request. In addition, 332 women with basal TSH levels outside the reference range were excluded. Finally, a total of 1485 patients with basal TSH levels within the reference range were left in the study. The patients were divided into normal high TSH levels (2.5–4.0 mIU/L) and low TSH levels (0.25–4.0 mIU/L).

### 2.2. Laboratory Methods

#### 2.2.1. TSH Measurement

TSH was routinely tested as a part of a screen for thyroid diseases before the initiation of IUI treatment. Specifically, the samples were collected on the morning of the 2^nd^–4^th^ days of menstruation; samples were centrifuged at 3000 rpm for 10 minutes after at least half an hour. Then, the serum on the upper layer was used for analysis. The measurement was conducted on an Immulite 2000, Siemens. A daily internal quality control for TSH measurement was performed.

#### 2.2.2. Sperm Preparation

The sperm preparation from the semen of the husband or the donor was performed by discontinuous density gradient centrifugation. Briefly, as for AIH, after 2 to 7 days of abstinence, the fresh semen was collected from the husband on the day of IUI, and for AID, the frozen semen was warmed in an incubator at 37 degree Celsius for 10 minutes on the day of IUI. When liquefied, the semen was placed on a liquid consisting of 45% and 90% gradients. Then, the semen was centrifuged at the centrifugal force of 300*g* for 20 minutes. Ideally, only the live sperm could reach the bottom of the centrifugal tube. Then, the semen plasma and gradients on the upper layer were removed. A five-minute washing followed, and the washing liquid was removed. Finally, the bottom sperm were transferred into the insemination liquid in a volume of 0.5 ml and a concentration of at least 20 million per microliter.

### 2.3. IUI Protocols

#### 2.3.1. Choice of Ovulation Scheme

Either a natural cycle or a stimulated cycle was applied to all the women based on the medical criteria. In the natural cycle, the patients had to have regular menstruation and previously normally developed follicles. The follicular development was assessed from the 2^nd^∼3^rd^ days of menstruation by transvaginal ultrasound. When dominant follicles (*d* > 10 mm) appeared after the tenth day of menstruation, the patients were asked to prepare for insemination. In the stimulated cycle, the patients had to meet one of the following diagnoses: PCOS, prolonged menstruation, a history of follicular dysplasia, failure of IUI with a natural cycle 2 or 3 times, and unilateral oviduct obstruction. The follicular development was assessed from the 2^nd^∼4^th^ days of menstruation. The patients were prescribed letrozole or clomiphene for 5 days if the pelvic cavity had no abnormal condition. When dominant follicles (*d* > 10 mm) appeared after the tenth day of menstruation, the patients were prepared for insemination. However, if the dominant follicle failed to develop, either HMG or FSH was prescribed at a dose of 37.5–75 IU per day until the follicles matured. For those with hypogonadotropic ovulation disorder, HMG was prescribed at the start of menstruation, and the doses were changed according to the development of follicle maturity.

#### 2.3.2. Timing of IUI

Either single insemination or double inseminations were performed when necessary. When the dominant follicle had a diameter greater than 15 mm, a daily transvaginal ultrasound was used to monitor follicle development until the average diameter was greater than 16 mm, endometrial thickness was no less than 7 mm, and urine LH was positive; then, HCG was injected at a dose of 10,000 IU, and the first IUI was conducted on the morning of this day. On the following morning, the rupture of the follicle was monitored by transvaginal ultrasound. If the follicle remained intact, another injection of HCG at a dose of 5,000 IU was performed. Then, the second IUI was conducted after ovulation. When the dominant follicle had a diameter greater than 20 mm, HCG with a dose of 10,000 IU was injected; the first IUI was performed on the following morning, and the second IUI was performed after ovulation. However, for the women with follicles that had already been ruptured before the first IUI or those with luteinized unruptured follicles after the first IUI, single insemination was applied.

#### 2.3.3. Statistical Analysis

Continuous data such as age, BMI, serum TSH value, and AMH value were expressed as the mean ± SD, and the statistical comparisons were performed by either Student's *t*-test or the Mann–Whitney *U* test. The chi-square test was used for the comparisons of categorical variables. Binary logistic regression was then used to evaluate the contribution of serum TSH level, age, BMI, infertile causes, and stimulated protocols on the miscarriage rate, clinical pregnancy rate, and implantation rate, which were set as serum *β*-HCG levels and verified as positive sixteen days after the IUI procedure.

Because the clinical pregnancy rate, implantation rate, and miscarriage rate between the AID scheme and AIH scheme were significantly different, the patients were categorized into the AID group and AIH groups based on TSH levels. Then, the abovementioned clinical outcomes were analyzed based on preconception TSH levels between 0.4 and 2.5 mIU/L and 2.5–4.0 mIU/L. Obstetric outcomes were analyzed based on TSH levels without consideration of sperm source.

## 3. Results

### 3.1. Characteristics and Distribution of Included Patients

The infertility duration of patients with normal high TSH levels was significantly longer than that of the low TSH level group (*P*=0.032) and more significantly longer in the AID group (*P*=0.005). The BMI was significantly greater in women with normal high TSH levels (*P*=0.030) and more significantly greater in the AIH group (*P*=0.016). However, female age, serum AMH level, VD-total level, FT3 level, FT4 level, and endometrial thickness were not significantly different. Moreover, the proportion of primary infertility, stimulation type, and causes of infertility of the normal high TSH level group were similar between both TSH level groups (Table 1).

It was reasonable to predict that the proportion of male factors leading to infertility was significantly higher, regardless of serum TSH levels, in the AID group. We found significantly fewer total PR sperm in infertility caused by male factors and infertility due to unspecified causes (56.62 ± 40.80 and 56.11 ± 45.81, respectively), which we believed to be male factors as well. However, because only those couples with a total number of progressive sperm greater than 10 million continued with IUI treatment, we found a slightly but not significantly higher progressive sperm number in clinical couples (76.72 ± 65.63 vs. 68.50 ± 52.79, *P*=0.17) (Table 2). As expected, the causes of infertility in the AIH group were similar in both TSH level groups. However, higher female age, partner age, higher AMH levels, and lower VD-total were found in the AIH group compared with those in the AID group regardless of TSH level.

### 3.2. Clinical Outcomes of IUI

The patients were categorized into the AID group and AIH group based on the final IUI scheme in light of the statistically higher pregnancy rate of the AID group, and then the clinical outcomes, such as the implantation rate, clinical pregnancy rate, and miscarriage rate, were compared among different TSH level groups. We found no significant difference between the normal high TSH level group and the low TSH level group when different conditions were considered (Table 3). The binary logistic regression revealed no impact of TSH levels on the implantation rate, clinical pregnancy rate, miscarriage rate, and live birth rate whether confounders such as female age, final IUI scheme, BMI, infertility duration, and serum AMH levels were adjusted or not. The odds ratios are shown in Table 4.

Of the 1485 women going through the first IUI, 329 women were found to present with fetal heartbeat in a gestational sac by ultrasound one month after insemination, while 36 women ended up with biochemical pregnancy. Of the 329 clinically pregnant women, 44 women suffered from miscarriage due to embryo arrest mostly, six women resulted in ectopic gestation, and 5 women unfortunately gave stillbirths. Among the 274 women who achieved live birth, 4 were excluded because of delivery of twins; then, the general obstetric outcomes were compared between the normal high TSH level and low TSH level groups, which are shown in Table 5, and it seemed that the TSH level had little impact on obstetric outcomes.

## 4. Discussion

SCH is characterized by elevated serum TSH levels and normal FT3 and FT4 levels. Krassas et al. [5] systemically analyzed the relationship between thyroid function and human reproduction and stated that the definition of SCH and included varied patients from different studies, which led to various affected population rates that ranged from 3.0% to 16.7% [6]. Levothyroxine supplementation was a safe and easy method for dealing with SCH and was reported to be associated with decreased risk of pregnancy loss and preterm birth, whether thyroid autoimmunity was present or not. Infertile women with thyroid autoimmunity sought help from ART to conceive of a healthy baby, and LT4 intervention seemed to have no improvement in pregnancy loss and preterm birth [7].

Infertile women with TSH levels from 2.5 to 4.0 mIU/L would be diagnosed with SCH when the definition of the upper limit is set as 2.5 mIU/L [8]. However, whether this population should be supplied with or without LT4 remains controversial. The guidelines published by the ASRM concluded that insufficient evidence showed that SCH was associated with miscarriage rate when the TSH level was between 2.5 and 4.0 mIU/L. We proposed that if there was no difference in clinical outcomes between infertile women with TSH levels between 2.5 and 4.0 mIU/L and below 2.5 mIU/L, why should we bother giving unnecessary treatment?

Several studies have been conducted to clarify this type of issue. However, different aspects were focused on such as a more specific TSH hierarchy and multiple IUI cycles [9, 10]. In this study, we selected patients undergoing their first IUI cycle to eliminate the possible alteration of TSH caused by ovarian stimulation [11]. We divided the patients based on serum TSH levels into two groups. It must be clarified, in the context of various TSH reference intervals present according to different instruments, that serum TSH measurement in this study was performed on an Immulite 2000, Siemens, the reference interval of which was 0.4–4.0 mIU/L.

In this study, we excluded patients with TSH levels out of the reference interval. To better illustrate the role of TSH levels in predicting the clinical outcomes, the general parameters of included patients between the high TSH level and low TSH level groups were compared. In the AID group, we found slightly longer infertility duration in women with high TSH levels, with a statistically significant difference, while in the AIH group, a greater BMI value was found in women with high TSH levels. As few studies have reported a comparison of female age between women undergoing AID-IUI and AIH-IUI, we further observed a much younger age (low TSH level group 29.26 ± 4.4 vs. 31.01 ± 4.56, *P* < 0.001; high TSH level groups: 28.87 ± 4.31 vs. 30.82 ± 4.36, *P* < 0.001) in women with AID-IUI, which may partially explain the higher clinical pregnancy rate in the AID group. Serum AMH levels showed the same trend as female age (low TSH level groups, *P*=0.031; high TSH level groups *P*=0.013), while serum VD-total levels showed the opposite trend in the high TSH level groups only (*P* < 0.001). Even so, binary logistic regression showed that this parameter did not significantly alter the impact of TSH levels on the clinical pregnancy rate, implantation rate, and miscarriage rate.

Consistent with previous studies [12–14], we also found a similar obstetric outcome in terms of single live birth rate, stillbirth rate, ectopic gestation rate, birth length and birth weight, and duration of gestation between the high TSH level and low TSH level groups, which supported no extra intervention when the preconceptional serum TSH level of infertile women was between 2.5 and 4.0 mIU/L.

There were also some limitations in this study. First, this was a retrospective study, which lacked power by its nature. Second, we did not report the status of thyroid antibodies in infertile women, which might have a role in infertility and miscarriage. However, the existing data are controversial regarding whether thyroid antibodies are associated with infertility or adverse reproductive outcomes [12, 15]. Third, we did not include the dose and types of medicine used for ovulation in women with stimulated cycles. Finally, we failed to trace more detailed information about the infants born in this study.

Nevertheless, we included as many possible factors for analysis as possible to minimize the bias that may affect the power of the impacts of TSH on clinical outcomes. We found that serum TSH levels between 2.5 and 4.0 mIU/L did not significantly influence the clinical outcomes of infertile women undergoing the first cycle of IUI treatment compared to those of women with lower TSH levels.

## Figures and Tables

**Table 1:** The characteristics of the included infertile couples.

	TSH [0.4–2.5)^c^ (*n* = 850)	TSH [2.5–4.0)^c^ (*n* = 635)	*P*	AIH			AID		
				TSH [0.4–2.5)^c^ (*n* = 552)	TSH [2.5–4.0)^c^ (*n* = 409)	*P*	TSH [0.4–2.5)^c^ (*n* = 298)	TSH [2.5–4.0)^c^ (*n* = 226)	*P*
Female age (years)^a^	30.39 (4.58)	30.13 (4.44)	0.262	31.01 (4.56)	30.82 (4.36)	0.529	29.26 (4.4)	28.87 (4.31)	0.314
Partner age (years)^a^	30.58 (4.89)	30.32 (4.9)	0.309	31.22 (5.12)	31.12 (4.91)	0.748	29.38 (4.19)	28.87 (4.54)	0.179
BMI (kg/m^2^)^a^	23.12 (3.15)	23.59 (3.59)	0.030	23.12 (3.1)	23.63 (3.44)	0.016	23.12 (3.26)	23.53 (3.86)	0.187
AMH (ng/ml)^a^	5.24 (3.92)	5.03 (3.78)	0.306	5.45 (4.17)	5.3 (4.02)	0.593	3.84 (3.36)	4.53 (3.28)	0.295
VD-total (nmol/L)	32.32 (14.21)	32.52 (14.3)	0.791	31.83 (14.16)	30.82 (12.97)	0.258	33.22 (14.26)	35.67 (16.3)	0.070
Endometrial thickness (mm)^a^	5.99 (2.36)	6.02 (2.07)	0.803	5.94 (3.49)	5.84 (1.91)	0.477	6.07 (2.1)	6.34 (2.3)	0.163
Infertility duration (years)^a^	3.29 (2.32)	3.58 (2.65)	0.032	3.32 (2.11)	3.46 (2.09)	0.761	3.23 (2.68)	3.99 (3.41)	0.005
FT4 (pmol/L)^a^	15.47 (2.16)	15.47 (2.05)	0.806	15.5 (2.18)	15.55 (2.04)	0.738	15.41 (2.12)	15.27 (2.06)	0.453
FT3 (pmol/L)^a^	5.26 (0.69)	5.29 (0.71)	0.421	5.2 (0.7)	5.27 (0.76)	0.171	5.36 (0.65)	5.33 (0.6)	0.504
Infertility type (*n*)^b^									
Primary infertility	551 (64.82%)	424 (66.77%)	0.434	323 (58.51%)	239 (58.44%)	0.98	228 (76.51%)	185 (81.86%)	0.138
Secondary infertility	299 (35.18%)	211 (33.23%)		229 (41.49%)	170 (41.56%)		70 (23.49%)	41 (18.14%)	
Ovulation type (*n*)^b^									
Nature cycle	495 (58.24%)	363 (57.17&)	0.68	295 (53.44%)	214 (52.32%)	0.731	200 (67.11%)	149 (65.93%)	0.776
Stimulated cycle	355 (41.76%)	272 (42.83)		257 (46.56%)	195 (47.68%)		98 (32.89%)	77 (34.07)	
Times of insemination									
Single insemination	20 (2.35%)	7 (1.10%)	0.074	9 (1.63%)	4 (0.98%)	0.574	11 (3.69%)	3 (1.33%)	0.109
Double insemination	830 (97.65%)	628 (98.90%)		543 (98.37%)	405 (99.02%)		287 (96.31%)	223 (98.67%)	
Causes of infertility (*n*)^b^									
Female factors	221 (26.00%)	174 (27.40%)	0.716	217 (39.31%)	173 (42.30%)	0.12	4 (1.34%)	1 (0.44%)	0.406
Male factors	317 (37.29%)	230 (36.22%)		91 (16.49%)	52 (12.71%)		226 (75.84%)	178 (78.76%)	
Unexplained factors	194 (22.82%)	134 (21.10%)		194 (35.14%)	133 (32.52%)		0 (0)	1 (0.44%)	
Others	118 (13.88%)	97 (15.28)		50 (9.06%)	51 (12.47%)		68 (22.82)	46 (20.35%)	

^a^Data are expressed as the mean (±SD); ^b^data are expressed as *n* (%); c: mIU/L. AIH : artificial insemination by husband; AID : artificial insemination by donor.

**Table 2:** Sperm parameters for husband.

	*n*	Total progressive sperm (million)^a^	*P*
Clinical outcomes			
Pregnant	133 (18.84%)	76.72 ± 65.63	0.17
Nonpregnant	828 (86.16%)	68.50 ± 52.79	

Causes of infertility			
Female factors	389 (40.48%)	82.02 ± 69.13^b^	<0.001
Male factors	143 (14.88%)	46.62 ± 34.80	
Unexplained factors	328 (34.13%)	82.21 ± 63.98^b^	
Others	101 (10.51%)	56.11 ± 45.81	

^a^Data are expressed as the mean (±SD) and *n* (%); ^b^compared with “male factors” and “others,” respectively, *P* < 0.001.

**Table 3:** Clinical outcomes of IUI in patients with different TSH levels.

		AID					AIH				
		Natural cycle	Stimulated cycle	Primary infertility	Secondary infertility		Natural cycle	Stimulated cycle	Primary infertility	Secondary infertility
Implantation rate	TSH [0.4, 2.5)^a^	81/200	44/98	98/228	27/70	125/298	36/295	56/257	56/323	36/229	92/552
	TSH [2.5, 4.0)^a^	53/149	30/77	68/185	15/41	83/226	23/214	43/196	38/239	27/170	65/409
	*P*	0.324	0.43	0.199	0.835	0.242	0.613	0.949	0.652	0.965	0.791

Clinical pregnancy rate	TSH [0.4, 2.5)^a^	76/200	43/98	94/228	25/70	119/298	29/295	47/257	47/323	29/229	76/552
	TSH [2.5, 4.0)^a^	49/149	28/77	63/185	14/41	77/226	22/214	35/195	33/239	24/170	57/409
	*P*	0.349	0.315	0.135	0.867	0.173	0.876	0.926	0.803	0.672	0.94

Miscarriage rate	TSH [0.4, 2.5)^a^	10/76	6/43	13/94	3/25	16/119	6/29	6/47	5/47	7/29	12/76
	TSH [2.5, 4.0)^a^	3/49	3/28	4/63	2/14	6/77	7/22	5/35	8/33	4/24	12/57
	*P*	0.208	1.000	0.127	0.838	0.254	0.366	0.842	0.104	0.502	0.498

^a^mIU/L. AIH : artificial insemination by husband; AID : artificial insemination by donor.

**Table 4:** Adjusted ORs of different TSH levels on obstetric outcomes.

	*P*	Crucial OR	95% CI	*P*	Adjusted OR	95% CI
Implantation rate	0.325	1.128	0.887–1.434	0.180	1.188	0.923–1.529
Clinical pregnancy rate	0.399	1.113	0.868–1.428	0.238	1.171	0.901–1.522
Miscarriage rate	0.527	1.237	0.640–2.387	0.429	1.320	0.663–2.629

OR: odds ratio.

**Table 5:** Obstetric outcomes of infertile patients with different TSH levels.

	TSH [0.4–2.5)^c^	TSH [2.5–4.0)^c^	*P*
Single live birth (*n*, %)^a^	157 (80.51%)	113 (84.33%)	0.514
Birth length (cm)^a^	50.25 (1.78)	50.58 (1.99)	0.183
Birth weight (g)^a^	3400.18 (604.56)	3480.39 (484.93)	0.159
Duration of gestation (d)^a^	276.42 (10.01)	278.69 (12.31)	0.096
Twins (*n*, %)^b^	4 (2.05%)	0 (0)	—
Stillbirth (*n*, %)^b^	3 (1.54%)	2 (1.49%)	1.000
Ectopic gestation (*n*, %)^b^	3 (1.54%)	3 (2.24%)	0.694
Preterm birth (*n*, %)^b^	20 (12.74%)	7 (6.19%)	0.077
Full-term birth (*n*, %)^b^	137 (87.26%)	105 (92.92%)	0.132
Post-term birth (*n*, %)^b^	0 (0)	1 (0.88%)	—

^a^Data are expressed as the mean (±SD); ^b^data are expressed as *n* (%); ^c^mIU/L. AIH : artificial insemination by husband; AID : artificial insemination by donor.

## Data Availability

The original data are available to all readers upon request. Please contact the first author at yuchao1988@yeah.net for such supporting data.
